# Hearing Loss and Chiari Malformation Type I: A Scoping Review

**DOI:** 10.3390/diseases13100315

**Published:** 2025-09-25

**Authors:** Andrea Migliorelli, Marianna Manuelli, Chiara Bianchini, Francesco Stomeo, Stefano Pelucchi, Silvia Palma, Andrea Ciorba

**Affiliations:** 1 ENT & Audiology Unit, Department of Neurosciences, University Hospital of Ferrara, 44124 Ferrara, Italy; 2 ENT & Audiology Department, University of Modena and Reggio Emilia, 41121 Modena, Italy

**Keywords:** Chiari malformation, Chiari malformation type I, hearing loss, sensorineural hearing loss

## Abstract

Background/Objectives: Chiari malformation (CM) type I is an uncommon condition that can be associated with a variety of neurological and otoneurological symptoms, including sensorineural hearing loss. The aim of this paper is to analyze the association between type I CM and hearing loss. Methods: A review of the literature was performed using PubMed/MEDLINE, EMBASE, and Cochrane Library databases, according to PRISMA criteria for scoping review (from 2000 to April 2025). Results: A total of 8 articles and 139 patients with type I CM have been included; the majority of studies focused on women, with a mean age of 38.5 years (range: 10–44 years). In two cases, surgery was necessary for restoring normal hearing thresholds. Conclusions: To date, the pathophysiological mechanisms related to type I CM and hearing loss are not fully understood yet; further studies are necessary to clarify these features and to evaluate the correct management of these patients.

## 1. Introduction

Chiari malformation type I is a neurological and neuroradiological condition characterized by the downward displacement of the cerebellar tonsils through the foramen magnum [1]. Its symptomatic prevalence is estimated at approximately 1 in 1280 individuals; however, the exact proportion is difficult to determine, as many individuals remain asymptomatic or exhibit only mild clinical signs throughout their lives [2].

The term Chiari malformation (CM) refers to a group of cerebellar anomalies first described by Hans Chiari in 1891, and which are currently classified into six types [3,4]. Type I is characterized as the mildest form of the condition and is diagnosed when the cerebellar tonsils descend at least 5 mm below the foramen magnum [4]. However, the degree of tonsillar ectopia demonstrates only a weak correlation with the clinical severity of the disease [5]. A descent of less than 2 mm is considered within normal limits, whereas a protrusion between 2 and 5 mm is referred to as cerebellar ectopia, which is estimated to be present in approximately 14% of the asymptomatic population [6,7].

The initial presentation of type I CM may occur in childhood; however, the majority of cases report symptom onset in adulthood (typically between 30 and 50 years of age) [8]. Type I CM is often discovered incidentally via magnetic resonance imaging (MRI) performed to investigate headache or other neurological complaints [4]. Some patients exhibit a reduced posterior cranial fossa volume, while others present with tonsillar ectopia despite normal fossa size [8]. Genetic studies have indicated the possibility of a hereditary component, particularly in families with underdevelopment of the posterior fossa bones [4]. Syringomyelia is a prevalent comorbidity, observed in 30–70% of cases, and is significantly correlated with the degree of tonsillar ectopia [8]. At present, MRI is the gold standard for diagnosis, as cerebellar herniation can be promptly visualized and measured on sagittal T1-weighted sequences [1,9].

The most frequently reported symptom is headache, although other manifestations such as neck pain, paresthesias, limb weakness, ataxia, dizziness, and fatigue may also be present [10,11,12,13]. Surgical intervention is considered in cases of progressive or disabling symptoms, with available procedures including posterior fossa decompression with suboccipital craniectomy and cervical laminectomy [8].

Sensorineural hearing loss has been identified as a potential symptom associated with type I CM [14,15]. Sensorineural hearing loss is a clinically significant and highly disabling condition affecting both adults and children, with a heterogeneous etiology including genetic, acquired, sudden, and age-related (presbycusis) causes [16,17,18,19,20,21]. In type I CM patients, sensorineural hearing loss has been reported with a variable prevalence ranging from 10% to 56%, and in some cases, it may represent the initial symptom of the malformation [22].

To date, only a limited number of studies have explored the relationship between hearing loss and type I CM, and no recent review on the topic is currently available. Aim of this scoping review is to analyze the association between type I CM and hearing loss, particularly assessing its incidence, audiological characteristics, and clinical progression.

## 2. Materials and Methods

A detailed review of the English-language literature on CM type I and hearing loss was performed using PubMed/MEDLINE, EMBASE, and Cochrane Library databases. The search period was from 1 January 2000 to April 2025, with the aim of selecting the most recent studies. The terms used for the search were “Arnold-Chiari Malformation”, “Chiari I Malformation”, or “Chiari Type I” and “hearing loss”, “hearing impairment” or “deafness”.

For PubMed/MEDLINE, we also used the following terms: (“Hearing Loss” [Mesh] OR “hearing loss” [tiab] OR “hearing impairment” [tiab] OR hypoacusis [tiab] OR deafness [tiab]) AND (“Arnold-Chiari Malformation” [Mesh] OR “Chiari I malformation” [tiab] OR “Chiari type I” [tiab] OR “Arnold Chiari I” [tiab]).

**Figure 1:**
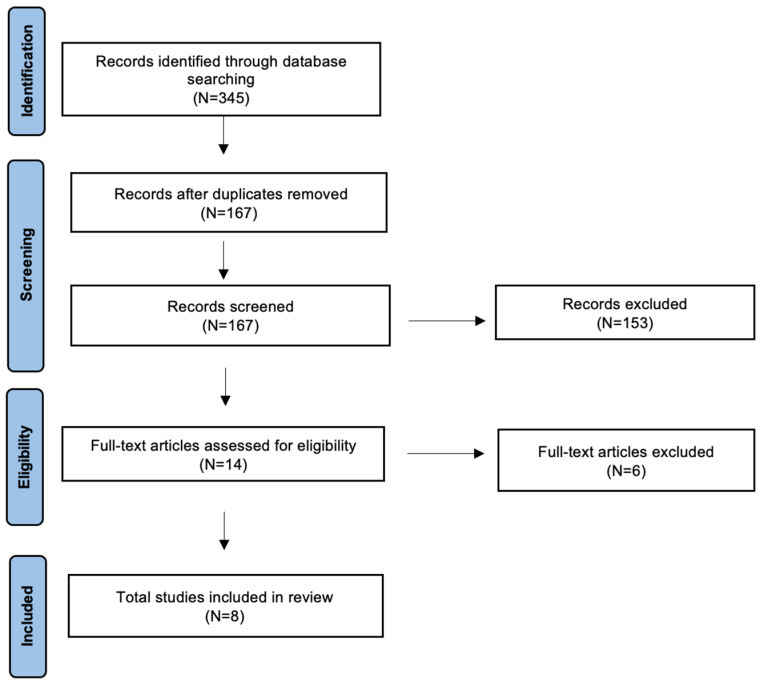
Preferred reporting items for systematic reviews and meta-analyses (PRISMA) graph.

The search yielded 345 candidate articles. The search was performed according to the “Preferred Reporting Items for Systematic Reviews and Meta-Analyses” (PRISMA) for scoping review guidelines (Figure 1) [23].

The inclusion criteria applied were: (i) publication date after 2000; (ii) clear description of the audiometric assessment; (iii) CM type I patients; and (iv) English language. Conference abstracts, publications written in languages other than English and articles in which the audiometric assessment was not clearly defined, have been excluded. Two authors (AM and MM) have evaluated independently all titles, and relevant articles have been individuated according to inclusion/exclusion criteria; a senior author (AC) resolved any disagreements. At the end of the full-text review, only 8 articles met the inclusion criteria [7,8,15,22,24,25,26,27].

For each article, the country of origin, the type of study, the number of patients with type I CM, and the total number of patients analyzed were evaluated. An evaluation was also conducted to consider gender and average age, onset symptoms, the presence of hearing loss, and its severity. Finally, an assessment of the surgical approach and the audiological outcome was also performed. The protocol for this review has been registered on the Open Science Framework (OSF) Registries with the Registration: osf.io/57dk9.

## 3. Results

The present review included eight articles, with a total of 139 patients with type I CM. Six of the studies were conducted in the United States, while two were undertaken in Turkey. The patients analyzed were predominantly female (93 females and 32 males), with a mean age of 38.5 years (range: 10–44 years). The predominant symptoms reported in the majority of studies were hearing loss and headache. Two articles analyzed patients who reported subjective hearing loss, but no abnormalities were identified in their audiometric tests [8,27].

The results of this review are summarized in Table 1. The analysis of the extant literature revealed that sensorineural hearing loss was the most commonly observed form of hearing impairment. The aforementioned symptoms were described in both unilateral and bilateral forms, with varying degrees of severity. The published literature does not report on conductive or mixed hearing loss. In three studies, patients underwent neurosurgical decompression, and in two of these, audiological outcomes were also reported, demonstrating a return to normal hearing thresholds. In the third study, however, no information was recorded on post-operative hearing thresholds. Finally, a case report documented the application of a pontocerebellar shunt, without subsequent audiometric improvement. The data concerning hearing loss and surgical intervention are outlined in Table 2.

### 3.1. Case Reports

Three of the studies analyzed are case reports. Dolgun et al. [25] reported on a case of a 44-year-old patient who presented with a history of headaches and a two-year history of bilateral hearing loss. Audiometric examination revealed moderate bilateral symmetrical sensorineural hearing loss. The MRI scan revealed type I CM with hydrocephalus and an arachnoid cyst in the left cerebellopontine angle. A left cerebellopontine shunt was performed. Audiometric testing conducted at the six-month postoperative stage revealed no substantial enhancement in comparison with the preoperative findings.

Heuer et al. [24] presented a case study of a patient diagnosed with type I CM at the age of 5, following an MRI for headaches. At the age of 10, the patient exhibited mild bilateral hearing loss, for which he underwent surgical decompression the following year. This procedure resulted in the restoration of normal hearing thresholds six months after surgery.

Finally, Sivakanthan et al. [26] analyzed the case of a patient with recurrent headaches and vomiting, where an MRI scan revealed CM. At the age of 18, the patient exhibited a sudden right hearing loss that did not respond to a steroidal treatment regimen. Consequently, the patient underwent a neurosurgical procedure involving decompression, resulting in the restoration of normal hearing acuity and the complete resolution of headaches within one year post-surgery.

### 3.2. Retrospective Study

Four of the eight studies analyzed are retrospective.

Haktanir et al. [7] conducted a study to evaluate the incidence of type I CM in 166 patients who underwent MRI scans for sensorineural hearing loss. The results were then compared with those of a control group of 50 healthy patients. The prevalence of CM in patients with sensorineural hearing loss was 7.2%, compared to 0.5% in the control group. The discrepancy between the hearing loss group and the control group was found to be statistically significant.

The objective of the study by Kumar et al. [15] was to report the clinical symptoms, neurological signs and results of vestibular tests and audiological assessments in 77 patients with Chiari I malformations confirmed by magnetic resonance imaging. Of the 77 patients, 35 presented with imbalance, 28 with occipital headaches, 25 with vertigo, 23 with sensory deficits in the upper limbs, and 21 with tinnitus. Audiometric evaluation was performed in 72 patients. In 40 patients, the audiogram was found to be normal. Twenty-two patients exhibited unilateral sensorineural hearing loss. The loss was categorized as mild to moderate in 14 patients and severe to profound in 8. In the remaining 10 patients, hearing loss was bilateral. In a total of nine patients, the loss was classified as mild to moderate, while in one patient, it was severe to profound. Consequently, sensorineural hearing loss was identified in 32 of the 72 patients analyzed.

Simons et al. [22] provided a detailed description of the prevalence of CM type I malformation in children referred to a tertiary pediatric center for hearing loss. In a study of 113 children who underwent magnetic resonance imaging as part of diagnostic tests for hearing loss, six cases of CM type I were identified. Of these six cases, four presented with unilateral hearing loss, one with bilateral asymmetric hearing loss, and one with bilateral symmetric hearing loss. All six children were referred to the neurosurgical department for further evaluation; however, it was determined that surgical decompression was not indicated in any of the cases.

Finally, Famili et al. [27] evaluated the audiometric tests of patients diagnosed with CM type I by MRI who complained of vertigo. The results of the audiometric tests indicated that 22 out of the 23 patients had results within the normal range; however, one patient exhibited a PTA of 21.25 dB HL in the left ear, accompanied by a history of noise exposure. Tympanometry was found to be normal bilaterally in all patients. Acoustic stapedial reflexes were present at expected levels, with no evidence of abnormal acoustic reflex decay in any of the subjects tested. Auditory brainstem responses (ABR) were recorded in 20 of the 24 patients. The analysis revealed that, with the exception of isolated prolongations in the absolute latency of wave III in the right ear (4.18 ms) and left ear (4.16 ms) of two individuals, all ABR data were within normal parameters. Consequently, auditory sensitivity and auditory brainstem function in the analyzed group were essentially normal.

### 3.3. Prospective Study

The present review incorporated a single prospective, non-randomized study. Sperling et al. [8] conducted audiological evaluations on a sample of 16 consecutive patients who subsequently underwent neurosurgical decompression of the posterior fossa. The most prevalent auditory symptoms reported were tinnitus and a sensation of fullness in the ear, both of which were observed in 81% of patients (13 out of 16). As was stated in the report, fluctuating hearing loss was experienced by a total of nine patients. Of these, six cases were bilateral, while the remaining three cases were unilateral. Eleven out of sixteen patients reported symptoms consistent with vertigo and nausea. The manifestation of auditory symptoms typically occurred concomitantly with the onset of headache episodes and was frequently ipsilateral to the side on which the headache was experienced. Five patients underwent audiometric evaluations, with normal hearing thresholds being demonstrated in almost all cases.

## 4. Discussion

In the CM type I, the medial and inferior portions of the cerebellar hemispheres (the cerebellar tonsils) herniate through the foramen magnum. This has been observed to be associated, albeit infrequently, with mild elongation of the fourth ventricle and medulla oblongata [28]. As the descent progresses, there is an impairment of cerebrospinal fluid (CSF) outflow, which often leads to the development of a tubular cavity in the upper spinal cord. Syringomyelia, a condition characterized by the formation of syrinx-like cavities within the spinal cord, occurs in approximately 50–70% of patients diagnosed with CM type I [29].

The present review shows that the majority of included patients were female, with an age range between 10 and 44 years. Typically, diagnosis is made using MRI, which clearly demonstrates the presence of cerebellar ectopia. The non-invasive nature and increasing availability of this technique have contributed to a notable rise in the number of imaging studies performed, thereby increasing the detection rate of incidental findings, including in asymptomatic individuals. In the entirety of the included studies, diagnosis was established using MRI [7,8,15,22,24,25,26,27].

The most frequently reported symptoms associated with type I CM are headache, nausea, vomiting, vertigo, imbalance, paresthesia, and neck pain. The majority of studies included in the present analysis identified headache as the symptom most frequently correlated with hearing loss [7,8,15,22,24,25,26,27]. Indeed, Sperling et al. [8] reported that fluctuating auditory symptoms were often associated with headache.

In a variable proportion of cases, sensorineural hearing loss has been observed to be among the symptoms associated with type I CM. In the present study, which was conducted on a sample of 139 patients, 54 (38.8%) of subjects presented with sensorineural hearing loss of varying degrees. It is important to note that the majority of the studies analyzed were conducted in highly specialized tertiary centers; therefore, the true incidence of sensorineural hearing loss of this disorder may be lower; since the present is a scoping review (not a systematic or a meta-analysis) it has not been possible to obtain type I CM prevalence data.

Hearing loss may present as the primary symptom or may manifest subsequent to diagnosis. The clinical presentation of this condition may be categorized as unilateral, bilateral (symmetric or asymmetric), and may range in severity from mild to profound [7,8,15,22,24,25,26,27].

It is also noteworthy that the majority of studies exploring the association between sensorineural hearing loss and type I CM originate from the United States, with a smaller number from Turkey [7,8,15,22,24,25,26,27]. Consequently, the findings may only be representative of a limited part of the global population. It is hoped that subsequent studies will encompass a more extensive geographic distribution.

A number of hypotheses have been advanced to explain the etiology of vestibulocochlear nerve dysfunction in CM type I patients. The following are included: (i) nerve traction due to brainstem herniation; (ii) compression of the cochlear nuclei or the eighth cranial nerve by the cerebellar tonsils; (iii) ischemic injury to the cochlear and vestibular nuclei resulting from microcirculatory disturbances or posterior inferior cerebellar artery compression; (iv) direct cochlear damage from elevated cerebrospinal fluid pressure transmitted through an anomalous cochlear aqueduct [24,30,31,32]. However, recent studies have not provided unequivocal confirmation of these mechanisms. Specifically, no significant differences in hearing loss severity (expressed in decibels) have been found between patients with cerebellar ectopia alone and those with fully diagnosed type I CM. These findings call into question the proposed pathophysiological mechanisms, particularly those involving nerve traction, nuclear compression, or vascular ischemia [7]. Haktanir et al. [7] performed a comprehensive analysis of vascular parameters and MRI was conducted, revealing no significant correlations between vessel diameters and the extent of hearing loss, with the exception of a positive correlation between the diameter of the left sigmoid sinus and cerebellar tonsil descent. This phenomenon may be attributed to the compression of the left sinus, precipitated by the crowding of adjacent extraparenchymal structures. Nevertheless, the absence of distinction between left and right tonsils may have constrained the interpretation of this finding. It is reasonable to hypothesize that ongoing technological advancements in MRI, including enhanced software and advanced acquisition techniques, will enhance the investigation of auditory pathophysiology in type I CM patients and support future research. Furthermore, the expanding implementation of radiomics and machine learning holds potential in assisting clinicians and radiologists in the management of these patients.

It is noteworthy that three of the included studies reported surgical decompression in patients diagnosed with type I CM [8,24,26]. Heuer et al. [24] performed surgical decompression in an 11-year-old patient to restore hearing function. The surgical procedure entailed a suboccipital craniectomy and a C1 laminectomy, during which tonsillar herniation was identified extending down to the lower portion of C2. The C1 laminectomy alone was sufficient to achieve decompression, obviating the necessity for tonsillar coagulation. Furthermore, an onlay dural cranioplasty was performed. Six months postoperatively, there was no longer any hearing loss, and audiometric thresholds had returned to within the normal range.

In a similar approach, Sivakanthan et al. [26] performed suboccipital decompression and C1 laminectomy on an 18-year-old patient with sudden-onset right-sided hearing loss who had previously been diagnosed with type I CM. A dural cranioplasty was also performed, once more without the necessity for tonsillar coagulation. The patient demonstrated a complete recovery, with hearing thresholds reverting to normal levels.

The suboccipital craniotomy is performed with the patient in a prone position and the head in a slightly flexed position. A skin incision is made down to the spinous process of C2, followed by layer-by-layer dissection and retraction of the cervical muscles to expose the posterior arch of C1 and the suboccipital bone. A suboccipital craniotomy is then performed, involving a wide opening of the foramen magnum and removal of the posterior arch of C1 (laminectomy) [26].

The surgical approach delineated in these case reports exhibits considerable potential in terms of auditory recovery [24,26]. However, to date, there has been a paucity of studies that have evaluated its real impact on auditory outcomes, and many questions remain regarding indications and optimal timing for the procedure. Consequently, it is anticipated that subsequent research involving larger patient cohorts will further examine the efficacy of suboccipital craniotomy with C1 laminectomy in enhancing hearing outcomes in type I CM patients.

In conclusion, sensorineural hearing loss appears to be frequently associated with Chiari malformation type I, although the underlying pathophysiological mechanisms remain poorly understood. Ongoing technological progress, particularly in neuroimaging, may facilitate future research and eventually elucidate these mechanisms. Despite being limited, the current data suggest that surgical intervention could significantly improve the hearing of selected patients. Further multicenter studies with standardized audiological protocols are necessary to improve our knowledge among the relationship between sensorineural hearing loss and type I CM.

Main drawbacks of this manuscript are: (i) the extreme variability in patient selection across different studies; (ii) the retrospective or case report nature of most studies; (iii) the limited number of studies and patients available; (iv) the time frame analyzed (interval 2000–2025), although it includes the most recent literature available, and the amount of papers published also matching the specific search, prior to year 2000, is very limited.

## 5. Conclusions

Sensorineural hearing loss is one of the possible clinical manifestations of Chiari malformation type I. This condition exhibits a wide spectrum of presentations and varying degrees of severity. In the present review, we found that more than 35% of type I CM patients reported sensorineural hearing loss.

Also considering the findings of this review, a multidisciplinary approach is essential in the management of type I CM patients. Specifically, all individuals diagnosed with type I CM should undergo regular audiological follow-up and MRI scans performed for audiological symptoms should include careful assessment of the cerebellar tonsils.

Further research is necessary to better understand the impact of surgical intervention on auditory recovery in this patient group, including the assessment of hearing outcomes through long-term longitudinal follow-up.

## Figures and Tables

**Table 1:** Literature review.

Author (Yrs)	Country	Type of Study	Nu CM	Aver. Yrs	Nu Adult/Nu Pediatric	Sex	Imaging Definition	Hearing Deafness	V	T	S or CPA	Other Symptoms
Sperling (2001) [8]	USA	P	16	41	16/0	F: 12 M: 4	>5 mm herniation	Y	Y	Y	0	Headache, nausea
Kumar (2002) [15]	USA	R	77	39	NA	F: 56 M: 21	>3- to 5 mm herniation	Y	Y	Y	13	Headache, neurological symptoms
Heuer (2008) [24]	USA	CR	1	10	0/1	F	NA	Y	N	N	0	Headache
Simons (2008) [22]	USA	R	6	4.9	0/6	M: 4 F: 2	>5 mm herniation	Y	N	N	0	N
Dolgun (2009) [25]	Turkey	CR	1	44	1/0	F	NA	Y	N	N	1	Headache
Haktanir (2013) [7]	Turkey	R	13	46.88	NA	NA	Ectopia: herniation more than 2 mm CM 1: herniation equal or more than 5 mm	Y	NA	NA	0	NA
Sivakanthan (2014) [26]	USA	CR	1	18	1/0	NA	NA	Y	N	N	0	Headache
Famili (2023) [27]	USA	R	24	40.88	24/0	F: 21 M: 3	>5 mm herniation	N	Y	Y	0	NA

Legend: Yrs: years; Nu: numbers, CM: Chiari Malformation Type I; Aver.: Average; P: prospective; R: retrospective; CR: case report; F: female; M: male; Y: yes; N: no; NA: not available, S: Syringomyelia, CPA: cerebellopontine angle disorders, V: Vertigo, T: Tinnitus.

**Table 2:** Audiological and surgical features.

Author (Yrs)	SNHL (Nu)	Audiometric Threshold/PTA SNHL Definition	Unilateral vs. Bilateral Definitions	Severity SNHL	Audiology Test	Surgery	Audiometric Surgical Outcome
Sperling (2001) [8]	N	25 dB	N	Normal	ENG, Ecog, ABR, OAE	Decompression	NA
Kumar (2002) [15]	Y (32) Unilateral (22) Bilateral (10)	NA	NA	Mild-Moderate: 23 Profound: 9	VFT	NA	NA
Heuer (2008) [24]	Y (1)	NA	NA	Mild	N	Decompression	Normal
Simons (2008) [22]	Y (6) Unilateral (4) Bilateral (2)	20 dB	Unilateral: hearing thresh- old of greater than 20 dB for at least 1 frequency (500–4000 Hz)	Mild: 1 Moderate: 3 Profound: 2	N	N	-
Dolgun (2009) [25]	Y (1)	NA	NA	Moderate	N	Shunt	Not improve
Haktanir (2013) [7]	Y (13)	20 dB (PTA: 250, 500, 1000, 2000, 3000, 4000, and 6000 Hz)	NA	NA	N	N	-
Sivakanthan (2014) [26]	Y (1) Unilateral	NA	NA	Profound	N	Decompression	Normal
Famili (2023) [27]	N	20 dB (PTA: 500, 1000, 2000, 4000 Hz)	NA	Normal	Tympanometry, ABR, Posturography, Rotational Assessment, VEMP	N	-

Legend: Yrs: years; Nu: numbers, Y: yes; N: no; NA: not available, SNHL: Sensorineural hearing loss, ENG: Electronystagmography, Ecog: electrocochleography, ABR: auditory-evoked brain response testing, OAE: otoacoustic emissions, VFT: vestibular function test, dB: Decibel, PTA: pure tone average, VEMP: Cervical and ocular vestibular evoked myogenic potentials.

## Data Availability

No new data were created or analyzed in this study. Data sharing is not applicable to this article.
